# Immunosuppressant Management in Renal Transplant Patients with COVID-19

**DOI:** 10.1155/2021/9318725

**Published:** 2021-10-14

**Authors:** Elham Ahmadian, Sepideh Zununi Vahed, Shakar Mammadova, Sima Abediazar

**Affiliations:** ^1^Kidney Research Center, Tabriz University of Medical Sciences, Tabriz, Iran; ^2^Department of Physical Geography, Baku State University, Baku, Azerbaijan

## Abstract

The coronavirus disease 2019 (COVID-19) pandemic poses a special risk for both immunosuppressed patients, especially transplant recipients. Although the knowledge about this infection is growing, many uncertainties remain, particularly regarding the kidney. Kidney transplant recipients (KDRs) should be considered immunocompromised hosts since a potential risk for infection, comorbidity, and immunosuppression exposure exists. Additionally, the management of immunosuppressive agents in KDRs remains challenging. Potential drug interactions with immunosuppressive treatment escalated the risk of unwanted side effects. In this review, we aimed to attain an augmented awareness and improved management immunosuppressant for COVID-19 KDRs.

## 1. Introduction

The coronavirus disease 2019 (COVID-19), caused by severe acute respiratory syndrome coronavirus-2 (SARS-CoV-2), was introduced as a serious pandemic by the World Health Organization (WHO) on 11th March 2020, although it had previously been detected in December 2019 in Wuhan City, Hubei Province, China [1]. The virus has phylogenic similarities with severe acute respiratory syndrome coronavirus (SARS) and Middle East respiratory syndrome coronavirus (MERS) [2].

Though the clinical manifestations are similar to other common respiratory viruses, the course might turn into a potentially life-threatening respiratory distress, multiorgan damage, or even a rapid death. It can end up with several other disorders, which generally influence the neural and gastrointestinal systems. Accordingly, older patients and individuals with comorbid situations such as diabetes, hypertension, cancer, chronic kidney diseases (CKD), immunosuppression, and renal replacement therapies are at high risk of severe infections [3]. Kidney transplant recipients are immunocompromised hosts of COVID-19 with higher risk of comorbidity due to prepresent CKD and long-term immunosuppression therapy. During both SARS and MERS epidemics, various solid organ transplantation recipients, including the kidney, died [4–6]. Also, the occurrence of inflammatory response, tissue damages, graft rejection, and suppression of the immune system can surge the risk of viral infections after organ transplantation [7, 8].

Due to the importance of immunosuppressive therapy in the new outbreak, here, we discuss COVID-19 immunosuppression management in kidney transplant patients.

## 2. COVID-19 and the Use of Immunosuppressants

The effect of immunosuppressant drugs on COVID-19 infection has been investigated which mainly incorporate corticosteroids, tocilizumab, and mycophenolic acid (MPA). In vitro studies have shown the growth inhibitory effects of MPA on SARS-CoV-2 [2]. Currently, the guidelines recommend the reduction and/or withdrawal of MPA in COVID-19 patients [2]. However, the results of recent studies might alter this approach.

It has been evidenced during clinical studies that corticosteroids are advantageous in COVID-19 infection, in particular in alleviation of the cytokine storm. Dexamethasone was shown to decrease mortality rate and diminish the need for hospitalization and the use of mechanical ventilation in a randomized clinical trial [9]. The majority of severely ill patients benefited from dexamethasone. Also, the results of different cohort studies indicated better clinical outcomes in patients treated with steroids [10–12] although two cohort studies have reported contradictory results [13, 14]. However, other studies with high validity and generalizability are required to confirm the effect of steroids in COVID-19 patients. Moreover, the effects of steroids on the host immune response against the virus should be clarified since the sufficient immune response during the initial phase of the disease can prohibit the progression of the infection [15]. Controversial data have been reported regarding the association between the use of steroids and SARS-CoV-2 clearance [16, 17].

The inhibitors of IL-6 (mainly tocilizumab), which have been connected with lower rate of mortality and ICU admission have gained great interest in the treatment of severe COVID-19 patients [18, 19]. However, again controversial results have been observed [20, 21]. Also, there is a high risk of confounding in these experiments. The efficacy and safety of tocilizumab in the treatment of COVID-19 have been assessed in some clinical trials [22, 23].

Currently, few studies are investigating the effect of immunosuppressants on COVID-19. However, the similarities between SARS and MERS with COVID-19 might pave the way in the management of immunosuppressants during this pandemic situation. Immunosuppressants such as calcinurine inhibitors (CNIs) and the mechanistic target of rapamycin (mTOR) inhibitors have shown inhibitory effects on the replication of MERS-CoV and SARS-CoV in vitro and thus might also be beneficial in COVID-19 [2].  Table 1 represents different classes of immunosuppressant, their mode of action, and immunological outcomes in COVID-19.

## 3. COVID-19 and Kidney Transplantation

### 3.1. General Considerations in Kidney Transplant Patients

The clinical manifestations, therapeutical options, and prognosis of COVID-19 in kidney transplant recipients would differ from normal population due to their special immunosuppressed status. Additionally, early diagnosis of the infection in these patients has great importance. Although lymphopenia is observed in the majority of general COVID-19 patients, it does not assist physicians in kidney transplant recipients since a preceding drug-related lymphopenia exists in these patients. Thus, exact monitoring of immunosuppressed patients and early detection of infection with suitable methods is pivotal. Zou et al. published the first case of COVID-19 infection in a kidney transplant recipient whose clinical signs were similar to other COVID-19 patients [24]. However, the progress of the infection in kidney transplant recipients has not been documented leading to missed information regarding the epidemiological features of COVID-19 in solid organ transplant patients. Although SARS and MERS outbreaks have been observed in transplant recipients, donor-recipient transmission has not been reported in COVID-19 so far [4, 6]. The latter might be affected by the exposure of the donor, asymptomatic patients, and infection during the incubation period. Also, the extent of infection and the duration of virus viability in organ compartments or blood may influence donor transmission risk [25]. Tight connections between transplant centers and infectious disease specialist as well as representing strict policies to report suspected COVID-19 cases could help professionals in order to acquire maximum protection.

The development of renal transplantation to an advanced clinical discipline with outstanding results is mainly in connection with the progress in immunosuppression. The current immunosuppressive guidelines are designed to hamper drug-related adverse effects mainly in synergistic regimens which allows reduction of immunosuppressive agent's doses; thus, decreasing side effects via simultaneous preserving the efficacy of the prescribed drugs. Enhanced efficacy and tolerability is obtained using current recommendations in immunosuppressive regimen protocols [26].

A one-size-fits-all practice does not exist for the management of immunosuppression in all cases. Several factors effect selection of a given regimen; the chief aim is induction of equilibrium between the benefit of rejection inhibition and over-immunosuppression risk. Current standard-of-care treatments have been extensively involved by transplant clinicians for more than ten years, whereas interest in newer drug opportunities remains muted and indefinite. It is believed that the selection of a regime should be guided by overall efficacy as well as immunological risks in individual subpopulations [27].

Different immunosuppressant agents have been introduced to be used in entered organ transplantation since 1995: tacrolimus, the mTOR-inhibitors, and mycophenolate. Currently, CNIs are considered, particularly in de novo transplantation, as the most effective maintenance therapy against acute rejection. CNI with mycophenolate or mTOR-inhibitor combinations has can hamper CNI dose and reduce nephrotoxicity. Uniform treatment regimens are suitable but should be set to individual cases. Extended follow-up investigation are required to select on the optimum maintenance therapy [28] .

### 3.2. Immunosuppression Management in Kidney Transplant Patients with COVID-19

It remains challenging how to manage immunosuppressive agents in transplant recipients infected with COVID-19 ideally. It has been shown that cancer patients with clinical manifestation of neutropenia confront unfavorable outcomes [29], which highlight the role immune system in combating COVID-19. It is then possible that there is room for immunosuppression drop to aid decreasing the progression of COVID-19. Instead, however, it could be losing the potential positive impact of immunosuppressive agents in alleviating cytokine storm regulated systemic inflammatory response [30]. Indeed, the majority of therapeutical approaches in COVID-19 target the inflammatory system [31]. These may include interleukin-6 (IL-6) receptor antagonists, glucocorticoids, anticomplement-5 inhibitors, mTOR inhibitors, and immunoglobulins, which have primarily been utilized against allograft rejection [32–35].

As mentioned before, the first case of COVID-19 pneumonia in a kidney transplant recipient was published in 2020 [24]. In this case, the triple regimen of immunosuppressant (including MMF, TAC, and prednisone) was stopped and a nonspecific immunoglobulin plus methylprednisolone (40 mg/kg) was commenced. After the remittance of fever, TAC administration was reintroduced at half the dose despite the presence of radiographic pneumonia manifestation. Two weeks after the onset of clinical signs, microbiological and radiological tests were negative and thus the former dose of MMF and TAC was restarted [24]. So, considering the inadequate experience and possibility of multiorgan failure after torpid evolution, which requires respiratory support, the recommended immunosuppressive protocol is the temporary interruption of immunosuppressant and introduction of low dose methylprednisolone in order to both control viral infection and prohibit vital complications in kidney transplant recipients. Corticosteroids given at lower doses exert different advantageous effects in renal graft recipients due to their anti-inflammatory, immunomodulatory, and vascular specification through prohibition of proinflammatory cytokine production, preserving the permeability and integrity of endothelium, maintaining cellular hemostasis, and decreasing the white blood cell traffic [36]. The withdrawal of MMF and decreasing the dose of CNIs could be another alternative strategy in less severe cases [37].

Our group in Imam Reza Hospital of Tabriz University of Medical Sciences, as other medical centers throughout Iran follows the guidelines as below (Table 2). In case of mild infection symptoms and the possibility of a high risk of rejection (first two months after transplantation, patients with a history of more than one organ transplant, patients transplanted with a high-risk immunological reaction), the patient's immunosuppression regimen should continue as usual [38]. If the patient's immunological risk is low and immunosuppression can be reduced, it is recommended that antiproliferative immunosuppressants (mycophenolate, azathioprine) and mTOR inhibitors (urolimus, sirolimus) be discontinued. In patients with moderate to severe infection, antiproliferative immunosuppressants should be discontinued and hydrocortisone or other injectable corticosteroids might be replaced (Alberici, Delbarba et al. 2020). CNI (tacrolimus or cyclosporine) is recommended to be discontinued, and in case of high risk of rejection, minimal concentrations could be prescribed [2]. Although mTOR inhibitors have been shown antiviral effects on some viruses, such as cytomegalovirus (CMV) and poliomyelitis (BK), such effects have not been observed regarding the coronaviruses. In addition, patients with acute respiratory syndrome are at risk for bacterial infections and mTOR inhibitors also have pulmonary side effects. Therefore, with the available information, it is suggested that in a group of transplant patients with COVID-19 infection who are concerned about rejection due to high immunological risk, the CNI immunosuppressant regimen should be prescribed instead of mTOR inhibitors [10].

However, according to the results of published papers, it is not comprehensively vivid the dose and restart time of immunosuppressive agents, and each patients should be evaluated separately. As a general conception, reintroduction of calcinurine inhibitor at half dose after the negative microbial cultivation and/or PCR seems to be reasonable, and MMF could be resumed during the upcoming days if the patient is asymptomatic [39]. However, the professionals need more experience and time to represent the best guidelines in managing immunosuppressant in renal transplant recipients with COVID-19.

Looking into the experience of other countries, antimetabolite, mTOR inhibitors, and calcinurine inhibitors have been withdrawn in France [40]. In another study, the use of MMF and mTOR inhibitors was held in solid organ tissue recipients with COVID-19, while TAC doses were diminished [41]. The use of antimetabolites was held in 56% of patients in the University of Washington registry; however, no connection was observed between the extent of immunosuppression baseline and COVID-19 outcomes [42]. As collected from different studies, MMF is the most common modified (withdrawn and/or deducing the dose) immunosuppressant since this intervention could also be done in other viral infections such cytomegalovirus and BK virus [43]. Although there is yet no certain agreement, the maintenance of some degree of immunosuppressant might help protection in relation the inflammatory phase of the infection and further respiratory failure. Also, calcinurine inhibitors are among the immunosuppressive agents which have been held in different centers and have been replaced by prednisone, although dexamethasone has turned into a better option in hypoxemic patient after the result of the RECOVERY trial [9].

## 4. Immunological Responses in COVID-19 Patients Undergoing Immunosuppressive Treatment

Immunological responses include innate and adaptive immune responses. Alveolar macrophages, monocytes, neutrophils, and dendritic cells are key components of the innate immune response to COVID-19 infection [44]. Increasing evidence propose the suppression of the innate immune response by SARS-CoV-2 in the initial stages of the disease. The activation of the inflammatory cascade subsequent to the activation of the immune cells is crucial for viral control but might also result in tissue injury [45]. Toll-like receptors, which activate downstream pathways in connection with inflammatory genes, are key receptor in the recognition of the virus [46]. Similar downstream cascades are activated via cytosolic RNA receptors (RNRs) which sense the nucleic acids of RNA viruses [47]. Cell entry and pathogenesis of SARS-CoV-2 is due to the function of angiotensin converting enzyme 2 (ACE2) found on monocytes and macrophages [48]. Although SARS-CoV triggers the formation of proinflammatory cytokines but it is unable to productively replicate within macrophages and dendritic cell [49]. This process remains vague about SARS-CoV-2 [50]. The severity of COVID-19 has been proposed to be in connection with overactivation or sustained proinflammatory sate of monocyte and macrophages and in turn immune response disturbances [50]. The suppression of the innate immune response in early stages of COVID-19 inhibits the activation of adaptive immune response, especially in severe cases. Lymphopenia is currently considered to be a sign on moderate and severe disease while mild infection has been associated with T cell increase [51, 52].

Immune responses are assumed to be different in immunosuppressed patients. Although it might be expected that immunosuppression is commonly unfavorable for the course of the disease, but in can be different in COVID-19. Exacerbated immune response, which can result in tissue damages is an important problem in COVID-19 [53]. Thus, immunosuppressants could be beneficial via the prohibition of cytokine storm. It has been reported that the early inflammatory response is plummeted in transplanted patients and the increment of IL-6 can be prognostic factor of COVID-19 progression [54]. Controversial data has been published regarding the mortality rates in transplanted patients with COVID-19 [55, 56]. However, longer cohorts are required to draw a clear conclusion. The effects of different immunosuppressant drugs on immune responses should be addressed in the upcoming experiment. For instance, although antiviral effects of CNIs has been shown in SARS-CoV and MERS-CoV, little is known about the clinical course of the drug in transplanted patients with these viral infections [57]. The antiviral effects of mTOR inhibitors against cytomegalovirus have been applied as an orphan drug option [58].

The persistence of the virus might be provoked by immunosuppression. It has been shown that prolonged virus persistence has been observed in immunosuppressed patients needing mechanical ventilation [59]. The type of transplant can also influence the patients' susceptibility to COVID-19. In case of kidney transplantation, more severe, chiefly cardiovascular comorbidities might occur due to the long dialysis courses [60]. However, as a general consideration, there is no consistent pattern for SARS-CoV-2 infection under immunosuppression. Very mild to severe cases have been observed in immunosuppressed COVID-19 patients [61].

## 5. Pharmacokinetic of Immunosuppressant Agents in Renal Transplant Patients with COVID-19

The best strategy in using immunosuppressant in renal transplant patients with COVID-19 could be discontinued or reduction in dose to help virus clearance. Longer viral presence in MERS patients who received high-dose corticosteroids has been documented [62, 63]. Thus, it is assumed that a reduction in immunosuppression might best prohibit the extended viral presence as well as induction of a stepwise balance between graft rejection and immune reconstitution. Careful assessment of risk benefit is also crucial in the context of pharmacokinetic principles since drugs show potential interactions. For instance, mTOR inhibitors and calcinurine inhibitors are generally metabolized via cytochrome P450 enzyme 3A4 (CYP3A4), CYP3A5, and P-glycoprotein (P-gp). Prednisolone is also cleared by CYP3A4 [64]. On the other hand, the glucuronidation process through the activation of uridine diphosphate glucuronosyltransferase enzyme 1A9 (UGT1A9) and UGT2B7 is mainly involved the metabolism of MMF [65]. The rapid development of new antiviral pharmacological opportunities against COVID-19 introduces drugs that mostly inhibit CYP3A4 and P-gp, which increases drug interaction possibilities. In the cases of HIV, substantial dose reduction as well as prolonged dosing interval of calcinurine inhibitors has been done in kidney transplant recipients [66]. Cyclosporine and TAC administration has been performed in a microdosing method [66]. Generally, in the concurrent administration of CYP3A4 inhibitors and immunosuppressant agents the clinical response, markers of toxicity and drug level should be carefully monitored. Coadministration of strong CYP3A4 inhibitors with TAC, everolimus, sirolimus, and cyclosporine requires regular therapeutic drug monitoring (TDM). Moreover, combination of strong CYP3A4 inhibitors with prednisone requires dose reduction. The use of antivirals which block CYP3A4 and P-gp enzymes in patients receiving mTOR inhibitors need and immediate withdrawn of the immunosuppressant agents. Then, after the achievement of the subtherapeutic level of mTOR inhibitors, TAC and/or cyclosporine could be started in a microdosing manner. However, in the case of everolimus withdrawal, the start time for cyclosporine should not be too early since everolimus level might surge due to CYP/P-gp interaction. Chloroquine administration can escalate cyclosporine concentration up to threefold [67, 68]; however, this impact is not observed with TAC. The long half-life of chloroquine (up to 2 months) can result in potential drug interaction especially in kidney failure cases; thus, close TDM is needed. In addition, coadministration of chloroquine with TAC requires CTc-interval assessments, considering these pharmacokinetic aspects can aid professionals in selecting the best immunosuppressant and providing an effective, safe, and evidence-based care in kidney transplant patients with COVID-19.

## 6. Other Complications

Common blood complications between viral infections (coronavirus and CMV), immunosuppressive drugs (especially mycophenolate and mTOR inhibitors), and antiviral drugs (Valganciclovir) should be carefully monitored by evaluating the patient's blood count [69]. Due to the risk for arrhythmias following the administration of some COVID-19 antiviral drugs (chloroquine/hydroxychloroquine, lupinavir/ritonavir), especially in concomitant use with other drugs with this complication such as tacrolimus and electrolytes, the electrocardiogram of the patient should be monitored daily [70]. If the patient has COVID-19 infection with diarrhea, this complication may increase the blood concentration of tacrolimus [71]. It is recommended to monitor the blood level of this drug and adjust its dose and avoid routine administration of loperamide to patients with diarrhea [72]. Since increasing the concentration of CNI immunosuppressants antiviral drugs can interfere with antiviral drugs, especially lupinavir/ritonavir, acute complications of these immunosuppressants such as hypomagnesemia, acute renal failure, and neurological complications such as tremor should be monitored [73].

## 7. Conclusion

Longer-term follow-up of transplant recipients will be crucial. Survival, hospitalization period, ICU days, and mechanical ventilation requirement have been the factors mainly evaluated, but the focus should be on allograft outcomes and immunologic factors, including the prevalence of acute cellular rejection, antibody mediated rejection, and de novo donor-specific antibody in the aftermath of COVID-19, particularly in immunosuppressant hampered patients. Additionally, late secondary (bacterial, viral, and/or fungal) infection possibility in immunomodulatory or dexamethasone-treated patients should be evaluated. In summary, patients with COVID-19 pneumonia who had undergone kidney transplantation might exhibit an unfavorable disease course and a poor outcome; hospitalization is regularly needed. Thus, in order to have an impact on the prognosis of these patients, improved clinical managements of immunosuppressant drugs is highly required.

## Figures and Tables

**Table 1:** Immunosuppressants, their mode of action and immunological outcomes in COVID-19.

Immunosuppressant class	Examples	Mode of action	Immunological outcomes in COVID-19	Reference
Corticosteroids	Dexamethasone, prednisone	Inhibition of lymphocyte gene expression	Suppressing cytokine storm prohibition of proinflammatory cytokine production, preserving the permeability and integrity of endothelium	[74]
Antimetabolites	Mycophenolic acid, azathioprine	Blocking DNA replication	Diminished immune response in vitro	[75]
Calcinurine inhibitors	Cyclosporine, tacrolimus	Inhibition of lymphocyte signalling	Selective inhibition of cytokine production and function	[57]
mTOR inhibitors	Sirolimus, everolimus	Inhibition of mammalian target of rapamycin (mTOR)	Inducing and cell cycle arrest in lymphocytes	[76]
Biologics	IL-2 inhibitors (daclizumab), IL-6 inhibitors(tocilizumab)	Act as anticytokine antibodies	Inhibiting the production of cytokines and thus alleviation of cytokine storm	[77]

**Table 2:** Immunosuppressive protocol in kidney transplant patients with COVID-19 infection in Iran hospitals.

Kidney transplant patients with COVID-19	Immunosuppression management	References
Mild infection	(i) In case of high risk of transplant rejection, continue immunosuppressant with the minimum effective dose (ii) If possible, reduce immunosuppression by discontinuing anti-metabolite drugs (mycophenolate, azathioprine)/family of mTOR inhibitors and continue administration of prednisolone and CNI with minimal effective blood concentration (iii) In mycophenolate+ mTOR inhibitor receiving patients, replace mTOR inhibitor with CNIs	([10], [37], [2])
Moderate to severe infection	(i) Continue prednisolone regimen with stress dose or replace it with intravenous hydrocortisone in case of shock (ii) If possible, disconnect other immunosuppressant agents (iii) In case of high risk of transplant rejection, discontinue antimetabolite immunosuppressants (mycophenolate, azathioprine)/mTOR inhibitors and replace mTOR inhibitor to CNI with minimal effective blood concentration (iv) In lupinavir/ritonavir or atazanavir/ritonavir treaed patients, usually even with discontinuation of mTOR and CNI inhibitors, adequate blood concentration of these immunosuppressants due to the prohibition of their metabolism continues during the course of antiviral therapy	([50], [10], [38], [2])

## Data Availability

The data used to support the findings of this study are included in the article.
